# Finding the original mass: A machine learning model and its deployment for lithic scrapers

**DOI:** 10.1371/journal.pone.0327597

**Published:** 2025-07-28

**Authors:** Guillermo Bustos-Pérez

**Affiliations:** 1 Departament of Human Origins, Max Planck Institute for Evolutionary Anthropology, Leipzig, Germany; 2 Institut Català de Paleoecologia Humana i Evolució Social (IPHES-CERCA), Tarragona, Spain; 3 Universitat Rovira i Virgili, Departament d’Història i Història de l’Art, Tarragona, Spain; Sapienza University of Rome: Universita degli Studi di Roma La Sapienza, ITALY

## Abstract

Predicting the original mass of a retouched scraper has long been a major goal in lithic analysis. It is commonly linked to lithic technological organization of past societies along with notions of stone tool general morphology, standardization through the reduction process, use life, and site occupation patterns. In order to obtain a prediction of original stone tool mass, previous studies have focused on attributes that would remain constant or unaltered through retouch episodes. However, these approaches have provided limited success for predictions and have also remained untested in the framework of successive resharpening episodes. In the research presented here, a set of experimentally knapped flint flakes were successively resharpened as scraper types. After each resharpening episode, four attributes were recorded (scraper mass, height of retouch, maximum thickness and the GIUR index). Four machine learning models were trained using these variables in order to estimate the mass of the flake prior to any retouch. A Random Forest model provided the best results with an $r^{2}$ value of 0.97 when predicting original flake mass, and a $r^{2}$ value of 0.84 when predicting percentage of mass lost by retouch. The Random Forest model has been integrated into an open source and free to use Shiny app. This allows for the wide spread implementation of a highly precise machine learning model for predicting initial mass of flake blanks successively retouched into scrapers.

## 1. Introduction

Scrapers are some of the most common lithic implements among archaeological lithic assemblages. They are present from the first Oldowan stone tools [1–3] through to modern ethnographic studies of hunter gatherers [4–7]. The “reduction model” [8,9] suggests that some stone tools (including scrapers) can represent different stages of reuse and modification through retouch. When considering scrapers within the reduction model, an integral concept is that of curation. The initial definition of curation included a series of behavioral patterns related to provisioning strategies [10,11]. However, further authors included into curation behavioral strategies such as tool transport, utilization in a wide range of tasks, anticipated production, hafting, and recycling (after the original tool had been discarded). An alternative definition of curation was proposed by Shott [12,13], who defined curation as “the ratio of realized to potential utility” [14]. This new approach to the definition of curation has important implications for lithic analysis and the study of lithic technological organization since it transforms curation into a continuous variable [12]. Given this new definition, and in the framework of the reduction model, the amount of mass lost by a lithic artifact by reuse/resharpening will be equivalent to its ratio of curation. Both, absolute (mass in grams) and relative (percentage of mass lost), variables can be empirically calculated in experimental contexts and estimated in archaeological contexts.

The presence of scrapers and their curation relates to several aspects of the organization of lithic technology of past societies [15]. Amount of curation affects the shape of stone tools at the moment of their discard (thus affecting the morphological variability of stone tool assemblages observed through time). Amount of curation can also relate to raw material sources, with more curated artifacts coming from longer distances [16,17]. Curation also relates to the selection of technological products for more intensive retouch, or shifts in technological strategies of transport, thus informing about the cultural choices and patterns of past human groups [18,19]. Finally, curation also relates to tool use and use-wear analysis.

Because of these reasons, predicting original scraper mass is a major goal in lithic analysis. Thus far, two approaches are employed to estimate the reduction and curation undergone by a retouched artifact. The first approach uses estimations derived from direct measurements on retouch. This has led to the proposal of several indexes using different measurements, such as height of retouch, length of retouched edge or projection of the original angle [20–24]. These indices usually provide good correlation values with mass lost, but they are usually conditioned by flake morphology, direction of retouch, or tool type (laterally retouched scrapers, endscrapers, bifacial products, etc.) and each one uses different scale ranges.

The second approach aims at estimating original flake mass based on remaining flake features. This approach has the advantage of not being limited by tool type, direction of retouch or flake morphology. Initial work focused on controlled experiments of flake formation using different measures of flake platform (width, depth) and exterior platform angle (EPA) to estimate flake mass [25–29]. Some of the reasons to select these features were that they usually remain unaltered in most retouched artefacts. These controlled experiments provided strong explanatory power for the formation of flakes, with flake mass being predicted with an $r^{2}$ value above 0.8 [30,31]. However, when the same variables are used to predict mass of experimentally knapped flakes, the predictive power of the model diminishes significantly, with $r^{2}$ values dropping to 0.403 [32] 0.224 for the same retouched flakes), and 0.384 [33]. These results meant an important drawback since, as Dibble [34] states: *“controlled experiments, in spite of their elegance, objectivity and replicability, are only useful if the results obtained from them are directly applicable to archaeological materials”*.

To overcome the limitations from these results, three approaches are commonly undertaken:

a) Adding additional features as predictive variables. Commonly flake thickness is added [35,36], since it is widely considered to remain unaltered through the reduction process. Other variables, such as scar count or remaining amount of cortex, seem to improve the predictive power of models [37,30].
b) Applying new methods for measuring more accurately existing variables. Examples are the refinement on traditional manual measurements of platform [38], the use of digital photographs [39], or 3D scans for measuring platform [40,31].
c) Applying different families of transformations in order to favor the Gaussian distribution of values of predictors and flake mass, thus increasing the predictive power of most models. These transformations usually use the cubic root [36,32,33] or different logarithmic transformations [37,30,40,31,41,42].

Additionally, although more commonly in core reduction studies, some authors have suggested approaching reduction by the combination of multiple indexes in order to mitigate possible errors [8,43,44]. It can be considered that these additions and improvements have provided correlation values of original flake mass on scrapers which allow for comparisons at the assemblage level. However, estimations at the individual stone artifact level remain unsatisfactory with a limited application to archaeological cases. This is due to three main reasons. First, while most research explores extensively the prediction of mass through different variables (and their interactions), it is usually not considered in the frame of continuous resharpening process, and when it is tested in this framework (continuous or single episodes of retouch), results provide lower correlation values [31,41]. Second, while most research aims at estimating original flake mass, less research provides results of estimations of percentage of mass lost against actual percentage of mass lost during retouch, which is the key component of the curated concept [6,12]. Third, most archaeological research addressing the prediction of original flake/scraper mass result in equations which might be difficult to extrapolate and practically apply other archaeological assemblages [35,41,45]. Recently [37,30], the use of machine learning has allowed the implementation of feature selection (identification of how many and which variables are better for prediction) and new algorithms. However, it has also resulted in limited improvements of the correlation coefficient [30], indicating that a possible threshold limit for this approach is being reached.

A new framework is needed to overcome the limitations of previous models (absence of being tested in sequential experimentations, higher accuracy, and easy implementation for all lithic analysts) aimed at predicting original flake mass from scraper attributes and being able to reach the individual scraper level. In the present study 134 flakes were successively retouched, providing a dataset of 694 episodes of resharpening. After each retouch episode, a series of attributes were measured and used to train four machine learning models and the best performing model is further evaluated for issues of collinearity and implemented through a Shiny app “Original Scraper Mass Calculator v.1.0”, which allows the user to manually introduce the data from a scraper to estimate its original mass or to upload all data at once and download the results.

## 2. Materials and methods

### 2.1 Experimental sample

The analyzed sample consisted of 134 experimentally knapped flakes using hard hammer (Table 1). The raw material of hammerstones varied widely (quartz, quartzite, sandstone, and limestone), which allowed for a diverse range of morphologies and potential active percussion areas. The experimental sample is dominated by flakes with feather terminations (n = 121; 90.3%), followed by flakes with hinge terminations (n = 10; 7.46%).

**Fig 1:**
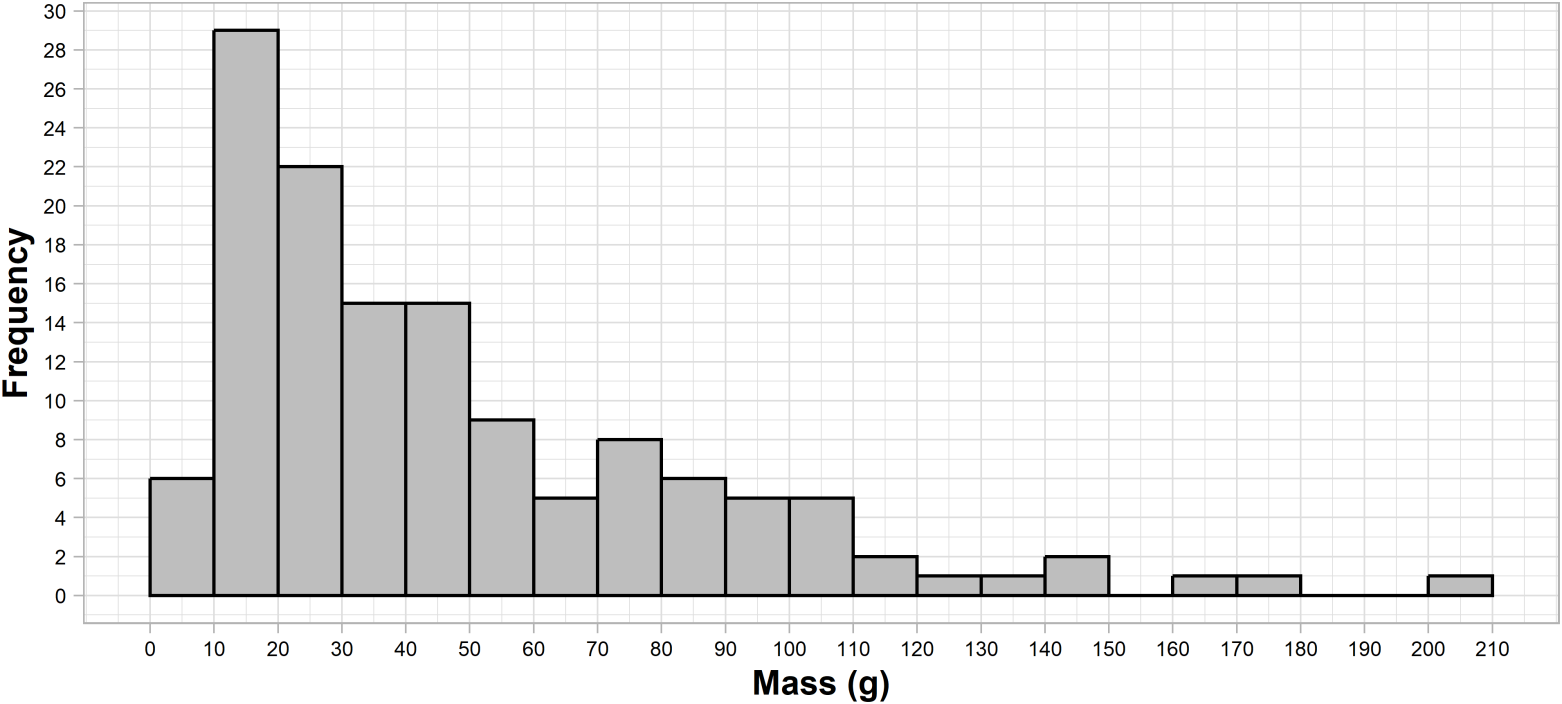
Histogram of mass distribution for the experimental sample of flakes.

Initial flake mass was recorded using a Sytech SY-BS502 with a precision of 0.01 grams. Average weight of the samples was 47.38 g, with 50% of flakes weighing between 18.07 and 63.16 g, and a standard deviation of 36.48. Fig 1 presents the flake mass distribution for the experimental sample, indicating a long tail of 14 flakes weighing more than 100 g.

The transversal section of flakes is considered to have an important effect on estimations derived from the geometric index of unifacial reduction (GIUR) [23] and height of retouch. In particular, when a flake’s dorsal surface is parallel to the ventral surface, the GIUR and height of retouch will only marginally or will not at all increase after each resharpening episode, resulting in underestimations of flake mass removal [8,22]. However, the actual effect of the *“flat flake problem”* on the estimation of flake mass might be marginal [46,47]. The present study recorded flake schematic transversal section prior to retouch of each flake, with possible categories being: circular (n = 20), triangular (n = 63), triangular asymmetric (n = 29), trapezoidal (n = 13) and trapezoidal asymmetric (n = 9). The first three categories are considered to represent cases where the “flat flake problem” is not present, while the latter two are consider to represent cases were this problem is present.

**Table 1:** Summary statistics of dimensions for the experimental sample.

Measure	Min.	1st.Qu.	Median	Mean	3rd.Qu.	Max	SD	CV
Length	24.5	50.95	62.60	63.23	75.80	106.70	16.90	0.27
Width	17.4	40.70	47.25	49.95	58.25	90.40	13.90	0.28
Middle Thickness	4.2	9.83	13.40	14.43	18.62	34.70	6.23	0.43
Maximum thick	6.0	12.03	15.80	16.57	20.00	36.00	6.31	0.38
Mean angle edge before retouch	13.5	33.08	39.84	41.27	48.67	72.33	12.32	0.30

All flakes were retouched until they were too small to hold while retouching (n = 4), they broke during retouch (n = 74), or the angle of retouch was too abrupt to detach additional resharpening flakes (n = 59). Retouch was done through freehand direct hard hammer on the dorsal face of flakes (direct retouch). In order to reshape the flakes one continuous retouched edge was established. After the first episode of resharpening, the retouched edge was expanded through the flakes edge in a continuous manner. This limits the potential application of this index to simple scrapers with direct retouch (which excludes double scrapers or scrapers with inverse or bifacial retouch).

Most flakes underwent between four and seven episodes of retouch (67.2%), while only seven flakes provided nine or more episodes of retouch. The experimental assemblage provided a total of 694 resharpening episodes with which to train the predictive models.

### 2.2 Feature selection

Based on previous research [23,36,37,47] variables were recorded as predictive features. After each episode of retouch, the following variables were recorded:

- Remaining scraper mass, recorded in grams with a Sytech SY-BS502 scale and a precision of 0.01 g. This variable is selected since machine learning models will consider remaining flake mass as a baseline on minimum mass of the scraper.
- Maximum thickness of the flake measured in mm (with a precision of 0.1). Feature selection through all possible combination of variables indicates that the logarithmic (base 10) transformation of this variable can increase the predictive power of regression models for freehand knapped flakes. Logarithmic transformation can result in Gaussian distribution of feature values increasing the predictive power of a model [37]. It is also considered that as resharpening proceeds, the thickness at the midpoint will be displaced (since length and width will diminish), while maximum thickness will remain more stable.
- Three equidistant measures of height of retouch (t) and the corresponding thickness (T) of the flake [47] measured in mm (with a precision of 0.1). The average of these three points is used as a predictive feature. Here it is considered that the average height of the retouch will serve as a proxy of mass removed from the scrapper.
- The GIUR index proposed by Kuhn [23]. This index divides the height of retouch (t) by its corresponding thickness (T). As previously indicated, the present study records three equidistant heights of retouch (t), each being divided by their corresponding flake thickness (T). The GIUR value is calculated as the average of these three divisions. GIUR values can range from 0 (unretouched flake) to 1 (when the height of the reaches the dorsal side of a flake).

The four variables were selected for training the regression models were: scraper mass, maximum thickness (log transformed), average height of retouch (t) and value of the GIUR index.

### 2.3 Regression models and evaluation

Four methods were employed for regression analysis: multiple linear regression, support vector regression with a linear kernel, random forest and gradient boosting machine.

Multiple linear regression (MLR) extends the simple linear regression in such a way that it can directly accommodate multiple predictors [48,49].

Support vector machines for regression [50,51] with a linear kernel (SVML) fit a linear hyperplane and a margin of error which allows for errors of points falling inside the margin. Points falling outside the margin define the support vectors. This provides a model focused on the general trend which aims to maximize the margin while minimizing the error, and which is also robust to the presence of outliers.

Random forest for regression selects random samples of the data and builds decision trees for prediction [52]. As a result, each tree is built from different combinations of the data, and the average is used as prediction. This adds diversity, reduces overfit, and provides high-accuracy predictions [53].

The gradient boosting machine (GBM) is an ensemble method that builds up a final model by incrementally improving an existing one [54,55]. The first model uses an initial *“shallow tree”* with a constant value (average of the labels). Following this initial model, a new tree (*weak learner*) is fitted to predict the residuals of the model, contributing to the final model predictions, allowing for correction of the errors of the model. This process is repeated, allowing it to progressively identify the shortcomings of week learners on a sequence of decision trees and to reduce the errors of the ensemble predictions.

Initial models are trained to estimate original scraper mass based on the set of selected attributes. However, as previously stated, the objective is to evaluate the ability of a model to predict the curation ratio of a stone tool (percentage of mass remaining relative to its original mass). Calculating the curation ratio of a stone tool can be formulated as:


100−((M/EOM)*100)

Where:

M = mass (directly measured on the scraper).

EOM = estimated original mass (provided by the model).

Models of both predictions (original scraper mass and curation ratio) are compared using four measures of performance: $r^{2}$, MAE, RMSE, and MAPE. $r^{2}$ is a measure of linear correlation and of how much of the observed variation is explained by the model [48]. In lithic studies, a categorization of the predictive power of indices has been proposed based on their $r^{2}$ values [56], where <0.1 is low, 0.1–0.25 is moderate, 0.26–0.5 is fairly large/strong, 0.51–0.8 is very large/strong and >0.8 is extremely large/strong [56]. However, it is important to consider that different distributions of data can result in same or similar $r^{2}$ values [57].

Mean average error (MAE), root mean squared error (RMSE), and mean average percentual error (MAPE) provide summary values of how far predictions fall from the true value [48,53]. MAE measures the average magnitude of errors, regardless of signal. RMSE also provides a measure of distance between predicted and actual values, although it punishes large errors. RMSE is usually compared to the standard deviation (SD) of the variable to be predicted. If RMSE presents a lower value than the SD, this is indicative of a good model which predicts values better than taking the average value of the sample. MAPE provides a measure of distance on a proportional basis (a residual of 3 g in a 12 g flake will be much higher than in a 50 g flake). A perfect model has a MAE, RMSE, and MAPE values of 0, and in general, better models will have lower values of MAE, RMSE, and MAPE.

Collinearity of the predictors is addressed through the variance inflation factor (VIF). VIF provides a measure of correlation between predictors and their effects on the model. In the present study VIF is calculated using the R package *car* v.3.1.2. [58]. Common thresholds for VIF values [59,60] range between 1–10 (considered inconsequential); 10–30 (cause for concern), and higher than 30 (seriously harmful). At present, the package *car* v.3.1.2 only allows for the calculation of the variance inflation factor for multiple linear regression. Although the different nature of the regression algorithms can result in different effects of collinearity, results from calculating the variance inflation factor in the multiple linear regression can be extrapolated to the rest of the models. Usually collinearity falls into two categories: data base collinearity (which results from the collection of data from the experiment) and structural collinearity (when new independent variables are generated from one or more existing predictive variables). Data base collinearity can be considered especially harmful, since collinearity present in the training dataset might not be present on new data on which to make predictions. However, since structural collinearity is the result of existing variables, it is expected to be present at training and unseen data. It is expected that the GIUR index and average height of retouch will present higher values of structural collinearity.

Models were evaluated using a k-fold cross validation. In k-fold cross validation, the dataset is randomly shuffled and divided into k folds. The first fold is employed as a test set, and the model is trained in the remaining folds. After this, the second fold is employed as a test set and the rest as a new training set. This process continues until all folds have served as a test set. Since the samples in each fold are determined by the initial random shuffle, it is advisable to repeat this cycle a series of times. The present work employs a 10-fold cross validation (six folds having a sample of 69 elements, and four folds having a sample of 70 elements) which is repeated 50 times with an initial random shuffling.

In addition to the above listed performance metrics, all models are graphically evaluated. A regression plot provides a scatter plot of predicted and true values along its regression line. In a good model, the regression line will pass through the center of all points, which will be evenly distributed above and below, and outliers are visible. Best performing model was selected based on metric performances values and visual evaluation of the regression plots.

The selected best model underwent additional testing to rule out possible overfitting due to the inclusion of multiple reshapening episodes, collinearity between variables, evaluate it’s ability to predict curation along the reduction process, original flake size, and to accuratly capture Weibull estimates and survival curves.

The evaluation of models using the k-fold cross validation was performed using the complete dataset of 698 resharpening episodes from the 134 flakes. This implies that, for a prediction, previous and posterior resharpening episodes of the same flake were included in the training data set. This raises the question whether the model is overfitting from seeing previous and posterior resharpening examples of the same flake. In order to evaluate this possible source of overfitting, a random selection of 10% (n = 13) of flakes and all their resharpening episodes were removed from the training set and the remaining sample used to train the previously selected best model. This process was repeated until 100000 predictions were obtained and then the four measures of performance were calculated. Additional to this, ten models were trained using only one resharpening episode per flake (training set = 134) and then used to predict on the remaining resharpening episodes (test set = 564). This allows to evaluate weather repeated values of original flake mass are resultingg in an overfitting of the best model. In both scenarios, a significant decrease in model performance would suggests overfitting, although a decrease in the predictive accuracy of the model is expected since significant proportion of information has been removed.

In order to further address the effect of collinearity among the best model, a principal component analysis (PCA) was performed on the four variables, and a new model was trained on the Principal Components (PC) adding up to more than 90% of variance. PCA is common dimensionality reduction technique which allows to eliminate collinearity between predictors, while minimizing the loss of information [61]. The distribution of predictions from both models are compared through Kolmogorov-Smirnov test. If collinearity between predictors is having causing overfit, the distribution of predicted values will be statistically significantly different. Additionally, two additional models excluding GIUR and average height of retouch (*t*) were trained and their performance metrics were compared in order to evaluate the possible redundancy of features.

The limitations of the best model are explored in five manners. First, the distribution of residuals (difference between predicted and actual values) is used in combination with transversal section of flakes to determine if the predictions are affected by the *flat flake problem*.

Second, the distribution of residuals from estimating curation was analyzed according to true curation ratio (in order to determine if the quality of predictions changes along the reduction sequence), according to original flake mass (to determine if a bias due to original flake mass is present), and according to predicted flake mass. Residuals of estimating flake mass compared to prediction of flake mass are also included. Residuals plots of a good model will have the points evenly distributed around the zero value across the range of the independent variables (Shapiro-Wilks test are performed in order to determine the normal distribution of residuals from estimating origina mass and curation ratio).

Third, different GIUR intervals are used to evaluate distribution of residuals. While original flake mass and actual curated ratio are appropriate variables to evaluate possible bias of the model, they are not truly known from the archaeological record. The GIUR value (and its intervals) can be obtained from archaeological scrapers and they can serve as a proxy on how reliable are predictions.

Fourth, curation ratios are evaluated per resharpening event, allowing to determine if over or underestimations are present. Finally, the Weibull distributions and survival curves of the experimental assemblage are fitted. Weibull distributions and survival curves are used to analyze the distribution of reduction among lithic assemblages [14,43,45,49,62–64]. Weibull distribution provides two parameters: β and η. The β parameter is indicative of the shape of the distribution, were a value bellow one indicates early discard, a value of one indicates constant discard, and a value above one indicates increasing failure with use. The η is indicative of the characteristic life/curation at which artifacts are discarded [62]. Survival curves of stone tools are classified into three types based on their shape [43,63]. Type I represents cases were discard occurs with greater us; Type II represent cases were discard is constant with its use; and Type III represents cases were discard occurs early. The present study fits and compares the Weibull distributions and survival curves of the actual and predicted curated ratios from the experimental sample.

Finally, two models were trained for comparison using the sample of 134 flakes. The first model uses the same set of variables from Bustos-Pérez & Baena Preysler [30] in order to predict original flake mass (log transformed). The second model used log transformations of platform size and maximum thickness along with exterior platform angle as predictive variables in order to predict the cubic root of flake mass. Both models were trained using a multiple linear regression, and predicted values were transformed to the linear scale in order to compare performance metrics with those of the best model from the current study.

The complete workflow was developed in RStudio IDE v.2024.04.02 [65] using the R programming language v.4.4.1 [66]. The package tidyverse v.2.0.0 [67] was employed for data manipulation and representation. Multiple linear regression uses the R package MASS v.7.3.60.2 [68], SVM with linear kernel uses package kernlab v.0.9.32 [69], the random forest model uses packages e1071 v.1.7.14 and ranger v.0.16.0 [69,70], and GBM uses package gbm v.2.2.2 [71]. Training and validation of models was done using the package caret v.6.0.94 [72]. Weibull distributions were fitted using package MASS v.7.3.60.2 [68], and survival curves were fitted using the survival v.3.8.3 package [73] and plotted using survminer package v.0.5.0 [74]. The “Original Scraper Mass Calculator” which implements the model through a user friendly interface was written using the shiny package v.1.8.1.1 [75–77]. All data, code is made publicly available through a public repository organized following the structure of a research compendium [78] and using a markdown document through package bookdown v.0.39 [79–81].

All data and the complete workflow of analysis is available as a research compendium [78] at Github (https://github.com/GuillermoBustosPerez/Scraper-Original-Mass/tree/main) and Zenodo (https://doi.org/10.5281/zenodo.15771459). The complete code and files of the Original Scraper Mass calculator v.1.0.0 is also available at Github (https://github.com/GuillermoBustosPerez/Original-Scraper-Mass-Calculator) and also at Zenodo (https://doi.org/10.5281/zenodo.15771479); and the final implementation of the application can be accessed at: https://guillermo-bustos-perez.shinyapps.io/Original-Scraper-Mass-Calculator/

## 3. Results

### 3.1 Resharpening effects on the experimental assemblage

**Fig 2:**
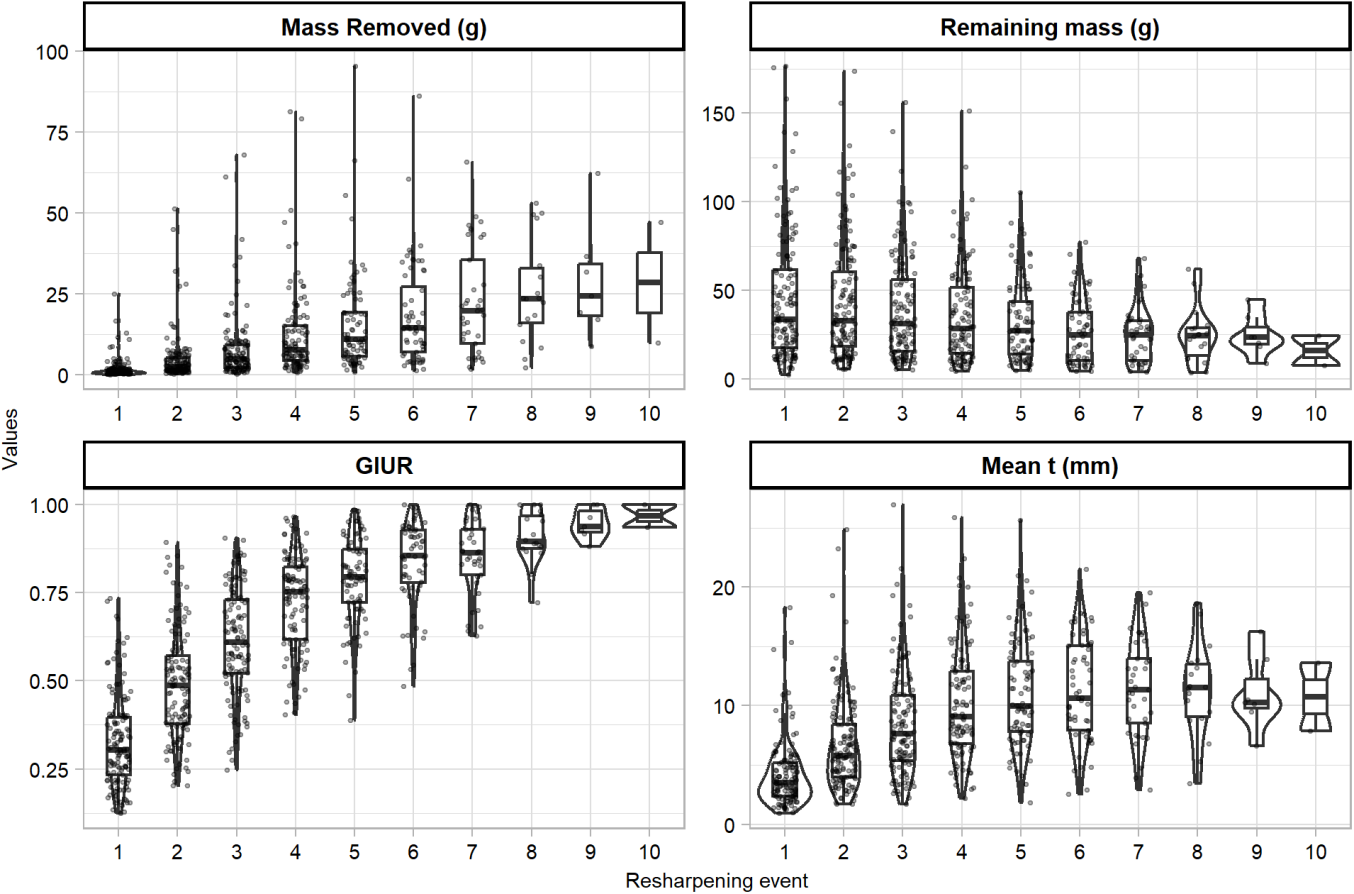
Box and violin plots of mass removed, remaining mass, GIUR index, and mean height of retouch (t), of the experimental assemblage according to each resharpening event.

Fig 2 presents the effects of each resharpening episode on the experimental assemblage. On average, flakes from the first episode of retouch had 1.84 g removed (with the exception of an outlier flake which had 25 g removed). Maximum value of mass removed was of 95.5 g after five episodes of retouch, while flakes reaching ten episodes of retouch had an average of 28.4 g removed. As for the retouched pieces, mass of the assemblage decreased from 45.9 g after the first episode of retouch to 15.9 g after ten episodes of retouch. On average, the resharpening episodes removed 20% (with a standard deviation of 15.9%) of mass from the scrapers. One resharpening episode removed a minimum of 0.513%, and a resharpening episode removed a maximum of 67.3% of mass. 50% of the resharpening episodes removed between 5.9% and 31.3% of mass.

In general, the GIUR remains fairly unidirectional, with increasing values after each episode of resharpening. The average GIUR after the first resharpening episode was 0.33. The first maximum GIUR value (1) was reached after six episodes of resharpening. Only six flakes of the experimental assemblage reached a GIUR values of 1, providing a total of eight different episodes. Mean height of retouch (t) increases during the first seven resharpening episodes, from an average value of 4.19 mm after the first episode, to a value of 11.4 mm after the seventh episodes. Average height of retouch (t) decreases progressively afterwards to a value of 10.8 mm on episode 10 of retouch. This decrease in average t is result of retouch reaching the upper dorsal side of flakes and diminishing the overall thickness.

**Fig 3:**
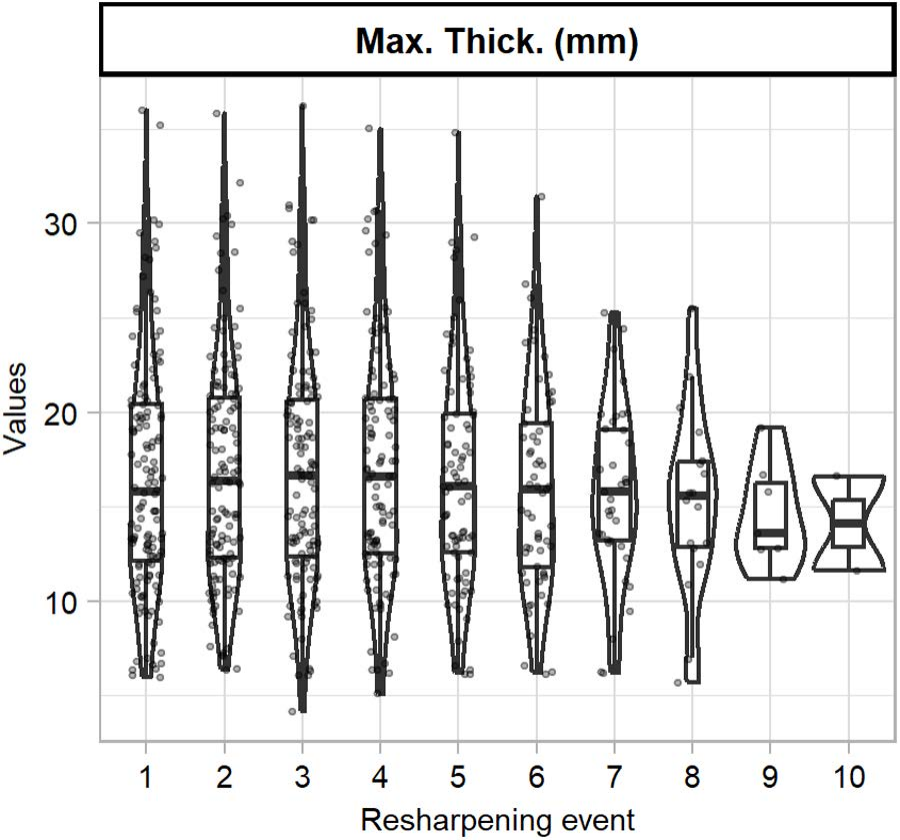
Box and violin plots of maximum thickness.

Maximum thickness (Fig 3) remained fairly constant among all the resharpening events, although the range of values decreases with each resharpening episode (this is due to a decrease in the number of flakes due to discard). After eight resharpening events, a light decrease in maximum thickness is observed due to retouch reaching the upper dorsal part of flakes, and reducing its overall thickness.

### 3.2 Evaluation of regression models

Table 2 presents performance metrics of each of the regression models for predicting original flake mass. All models had RMSE values lower than the standard deviation of original flake mass, indicating a good fit of the models. The random forest model presented the best values for all evaluation metrics, being able to capture 0.974 of variance, and with a MAE of 3.297 g. It also presented the lowest RMSE value (5.917), indicative of being the least affected by outliers in the predictions. The lowest MAPE value of 6.775 indicates that the random forest model is also the least affected by the size of the original blanks. The GBM model has evaluation metrics similar to those of random forest, being able to capture a similar proportion of variance (0.971) and slightly higher values of MAE (3.549) and RMSE (6.185). In general, multiple linear regression was the model with the worst performance metrics, capturing slightly more variance than the SVM (respective values of 0.963 and 0.961) and being less affected by the presence of outliers (respective values of 7.036 and 7.265).

**Table 2:** Performance metrics of regression models for predicting original flake mass.

Model	r^2^	MAE	RMSE	MAPE
Mult. Linear Reg.	0.963	4.672	7.036	12.269
SVM Linear	0.961	4.394	7.265	11.304
Random Forest	0.974	3.297	5.917	6.775
GBM	0.971	3.549	6.185	7.705

**Fig 4:**
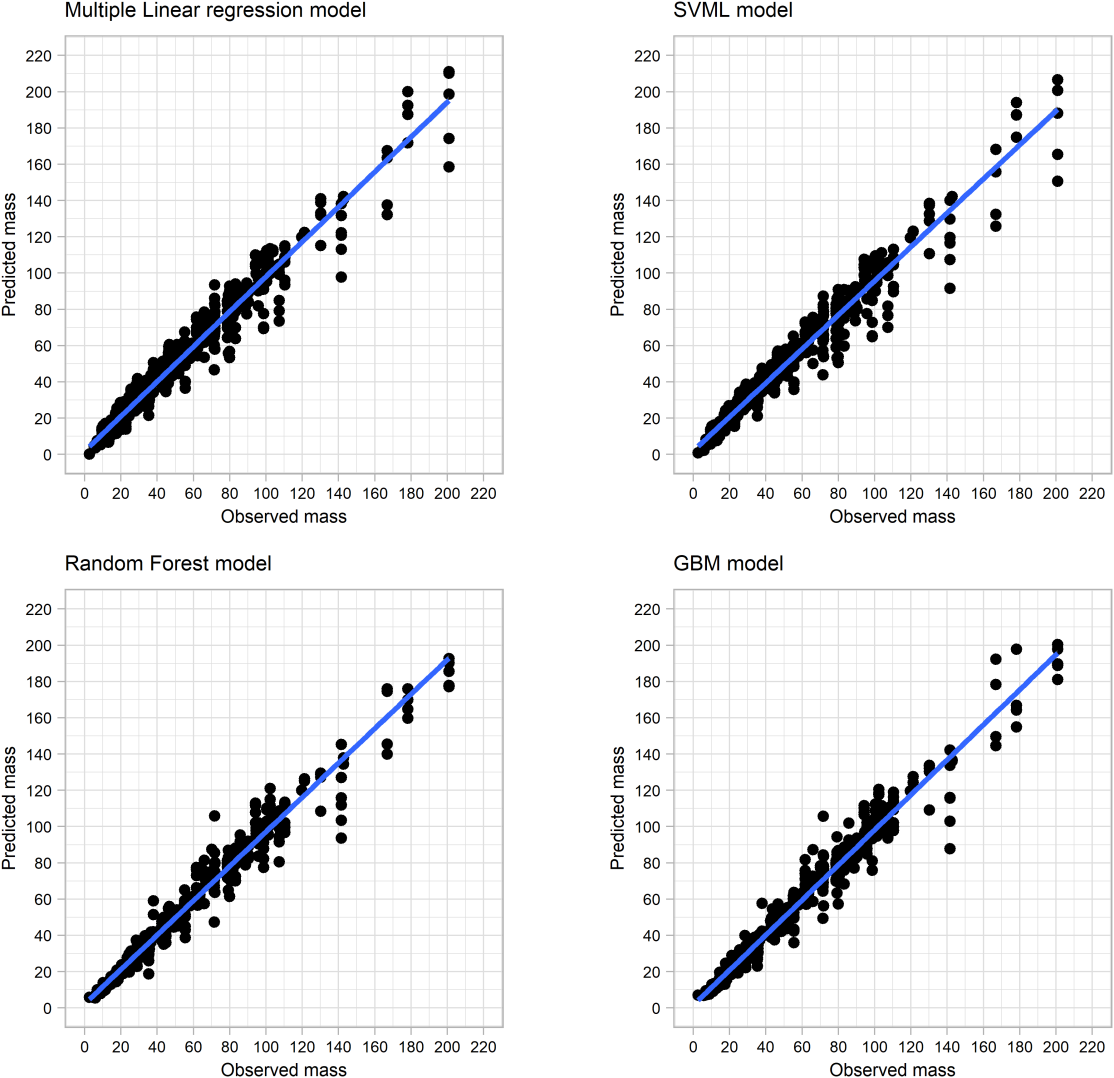
Regression plots of each of the trained models when predicting original flake mass.

Fig 4 presents the regression plots of all trained models. All models present regression lines with evenly distributed predictions, indicative of an absence of bias. However, it is observed for the multiple linear regression and SVM that with increasing flake mass, the range of predictions becomes more dispersed.

**Fig 5:**
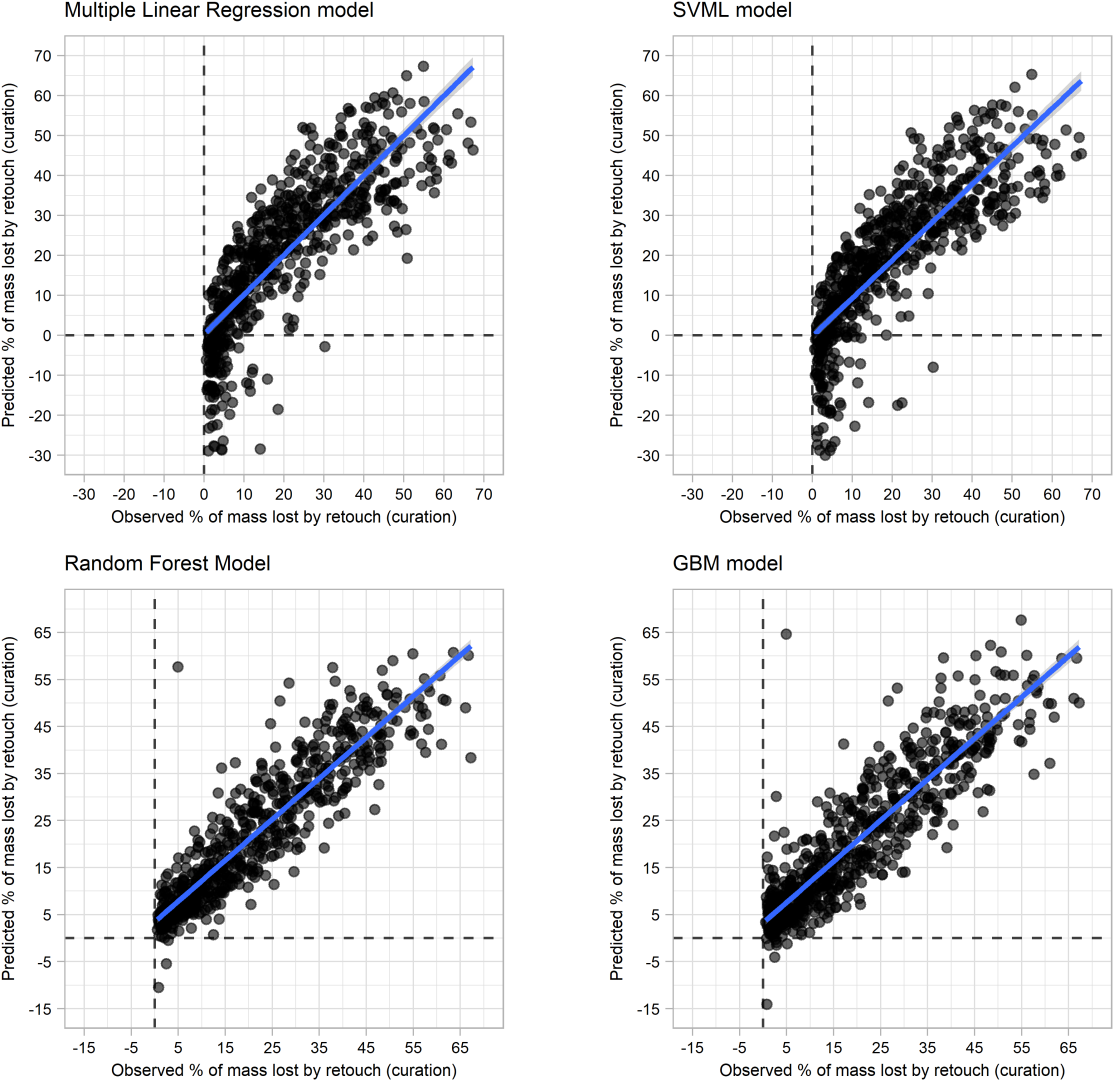
Regression plots of each of the models when predicting the curated ratio of each of the episodes of retouch.

Percentage of flake mass consumed by retouch (curation) was calculated using estimations of original flake mass for each model and each episode of resharpening. Table 3 presents performance metrics of the four models when predicting the curation ratio, and Fig 5 presents their corresponding regression plots. Important differences can be observed between models when predicting percentage of mass lost by retouch based on predictions of original mass. Of the four models, only random forest and GBM presented adequate performance metrics. Multiple linear regression presented the lowest performance values, with a linear correlation value of 0.113, while SVM with linear kernel presented a significant higher (but still unsatisfactory) $r^{2}$ value of 0.399. Both models also presented RMSE values higher than the standard deviation of percentage of mass lost through retouch (SD = 15.92). Visual evaluation of the regression plots indicates that the low performance metrics from both models (multiple linear regression and SVM with linear kernel) seem to be caused by underestimations original flake mass when a small percentage of flake mass has been removed by retouch. As a result, multiple linear regression estimated an original scraper mass value lower than remaining scraper mass in 127 cases. SVM with linear kernel estimated an original scraper mass lower than remaining scraper mass in 118 cases.

**Table 3:** Performance metrics of models when predicting percentage of mass lost by retouch (curation).

Model	r^2^	MAE	RMSE	MAPE
MLR	0.113	11.726	54.429	1.037
SVML	0.536	9.537	16.103	1.449
Random Forest	0.839	4.662	6.485	0.365
GBM	0.805	5.189	7.160	0.449

Linear correlation values indicate that random forest and GBM perform at least twice as well than the SVM with linear kernel, with respective $r^{2}$ values of 0.839 (random forest), and 0.805 (GBM). Both models also present RMSE values lower than the SD of the sample, indicative of a good fit of the model. The random forest model presents the best performance metrics, with the highest linear correlation value ($r^{2}$ = 0.839) and lowest values for MAE (4.662), RMSE (6.485) and MAPE (0.365). Random forest is closely followed by the GBM model, with a linear correlation value of 0.805. Visual evaluation of the regression plots (Fig 5) reinforces the notion of the good performance of random forest and GBM. In both cases prediction points are evenly distributed among the regression lines, and clustering of points in the lower values (due to over or underestimation of curation) are absent. GBM estimated an original scraper mass lower than the remaining scraper mass in 8 cases, while random forest made the same error in only three cases.

### 3.3 Bias, limitations of the best model (random forest) and comparison with other models

The episodes of resharpening generated 130 cases where the flat flake problem is observed and 564 cases where this problem is not observed. Student’s t-test comparing the residual distribution of flat and non-flat flakes shows no statistical differences (t = −1.93; p = 0.17) for all episodes of resharpening. When selecting flakes that had four or more resharpening episodes, no statistical significance is observed for the residual distribution (t = −0.79; p = 0.43). This indicates that for the given sample, and the given predictive variables, random forest predictions are not affected by the flat flake problem. A K-S showed no statistical difference between the distribution of observed and predicted original flake mass (D = 0.035; p = 0.8).

None of the predictive variables in the multiple linear regression model presented VIF values above the threshold of 10. Average height of retouch (t) presented the highest VIF value (7.27), followed by the GIUR index (4.59). Log transformed maximum thickness and remaining scraper mass presented respective VIF values of 3.81 and 2.35. PC1 (68.17%) and PC2 (26.55%) add up to 94.72% of variance and were selected to train a new Random Forest. When comparing the distribution of predictions from both models, no statistically significant difference was fount (D = 0.039; p-value = 0.67), indicating that the predictions from both models come from the same distribution, and that the original model is not affected by collinearity. Model training leaving out either GIUR index or average t resulted in similar performance metrics to those of the complete model. The performance metrics decreased slightly more when GIUR index was removed ($r^{2}$ = 0.969, MAE = 3.661; RMSE = 6.415, MAPE = 7.616), than when average height of retouch was removed ($r^{2}$ = 0.971, MAE = 3.556; RMSE = 6.237, MAPE = 7.177).

**Fig 6:**
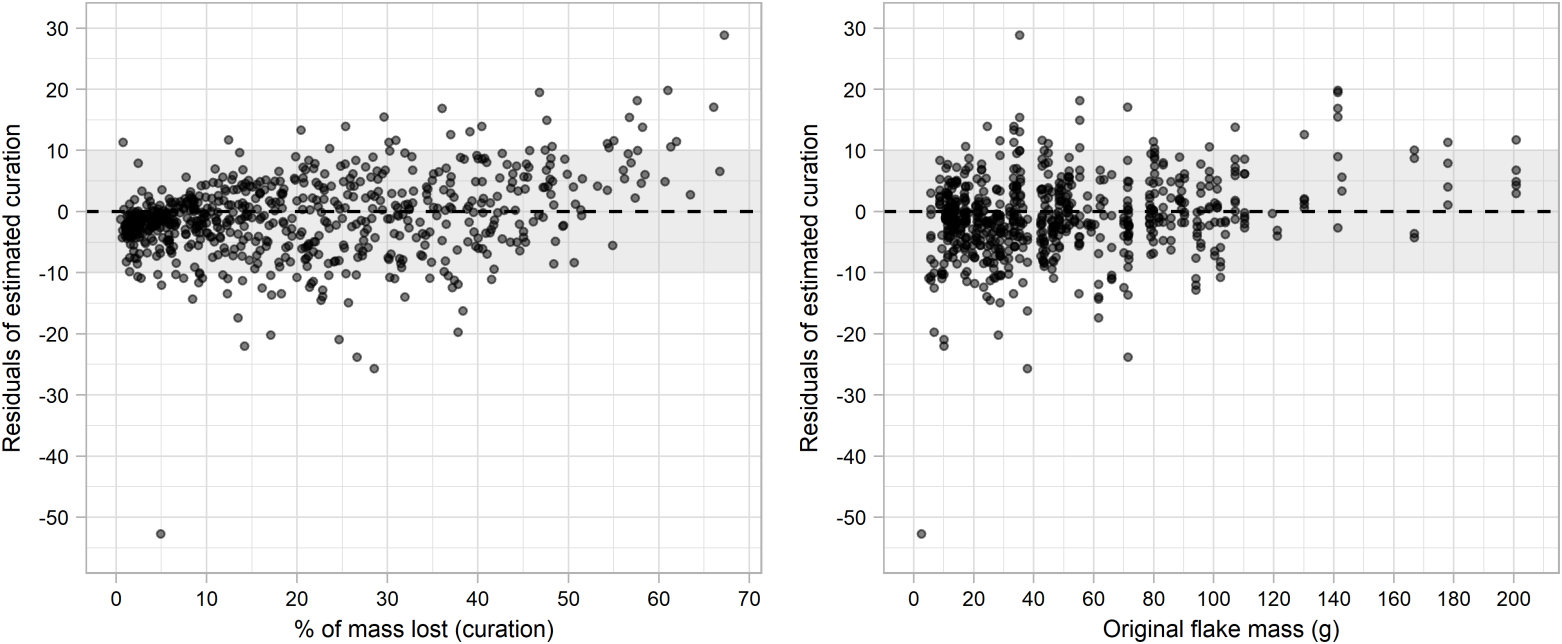
Residual distribution of estimated curation according to actual curation and original flake mass.

Fig 6 presents the distributions of curation residuals (difference between actual curation and estimated curation) according to real curation and original flake mass. Average value of curation residuals for the complete sample was of −0.94. Despite the presence of an extreme outlier (with a curation overprediction of 52.74%), only 75 episodes (10.81% of the resharpening episodes) showed cases were curation was over/under estimated more than 10%. Underprediction of curation seems to be more common when actual curation presents a value equal or above 55%. The dataset registered 31 cases were curation ratio was equal or higher than 55%, of which eleven cases (35.48%) presented an under/overestimation greater than 10%. When real curation is segmented into four intervals (Table 4) significant differences is present among the residual distribution of curation (Kruskal-Wallis test: chi-squared = 65.38, df = 3, p < 0.001), with respective average values being −2.12, −1.35, 1.04 and 7.81. Despite this, little difference is observed between the performance metrics of the first three intervals of curation Table 4.

**Table 4:** Performance metrics when predicting curation for each of the intervals.

Curation interval	Count	Mean error	MAE	RMSE	MAPE
(0.446,17.2]	364	−2.117	3.494	5.393	0.829
(17.2,33.9]	178	−1.354	5.687	7.172	0.235
(33.9,50.6]	122	1.042	5.637	6.976	0.138
(50.6,67.3]	30	7.816	8.791	10.809	0.150

**Fig 7:**
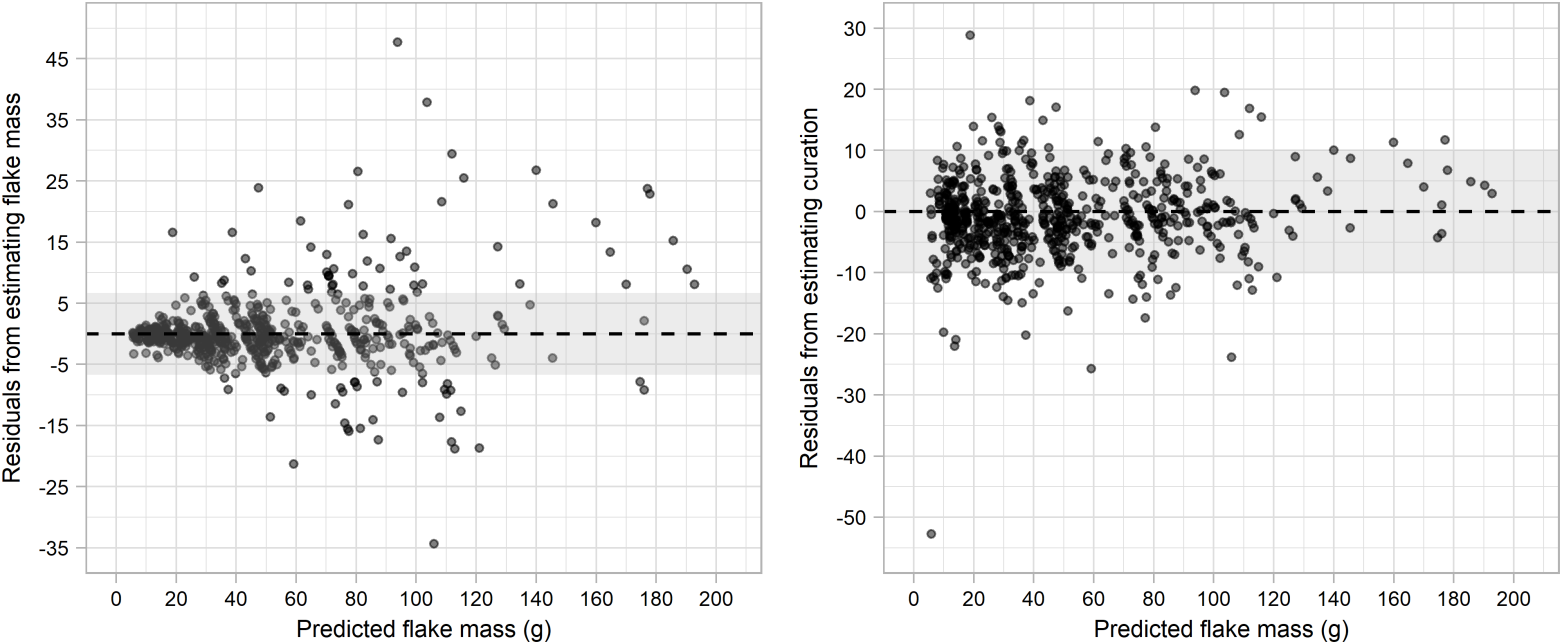
Residual distribution of estimated mass and curation ration according to predicted mass.

Fig 7 presents the distribution of residuals when estimating flake mass and curation according to predicted flake mass. In the first case it can be observed, that for a higher value of predicted flake mass, residuals will become more disperse along with an underestimation of flake mass. Results from parametric distribution indicates a non normal distribution of residuals from estimating original flake mass (W = 0.771; p < 0.001). Residuals of curation according to estimated original mass present a much more even distribution (Fig 7). Residuals are evenly distributed along the zero value, although the model will be prone to underpredict curation ration for flakes predicted to have a mass higher than 120g.

On general, flakes with an initial mass above 140g presented underestimations of original mass. The dataset provided 21 resharpening episodes of flakes with a mass value equal or higher than 140g. Of these 21 episodes, seven cases (33.33%) presented under/overestimations greater than 10%. When original flake mass is segmented into four intervals (2.44 to 52.2g; 52.2 to 102g; 102 to 151g; and 151 to 201g) significant differences is present among the residual distribution of curation (Kruskal-Wallis test: chi-squared = 26.9, df = 3, p < 0.001), with respective average values being −1.58, −0.52, 2.75 and 5.05.

**Fig 8:**
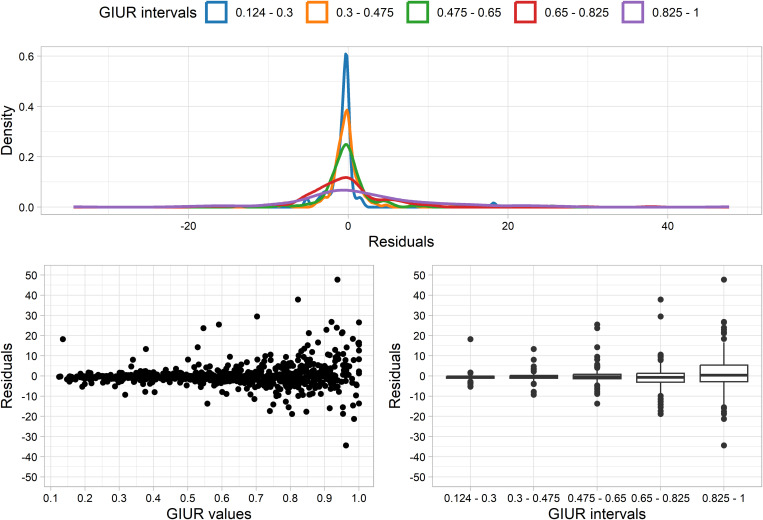
Residual analysis according to GIUR intervals and continuous values using density, scatter and boxplots, when predicting flake mass.

GIUR values can be used as proxies for quality of predictions. Fig 8 presents the residuals (when predicting original flake mass) distribution of continuous GIUR values and the same divided into five intervals. A density plot indicates that, on a general level, residuals tend to peak at the zero value. However, with increasing GIUR intervals the peak among the zero value diminishes. Scatter and box plots also indicate that, for higher GIUR values and intervals, a greater range of residual values is present. This indicates that, although residuals are evenly distributed around 0, the accuracy of predictions from the random forest diminishes among heavily retouched flakes (with GIUR values above 0.8).

**Fig 9:**
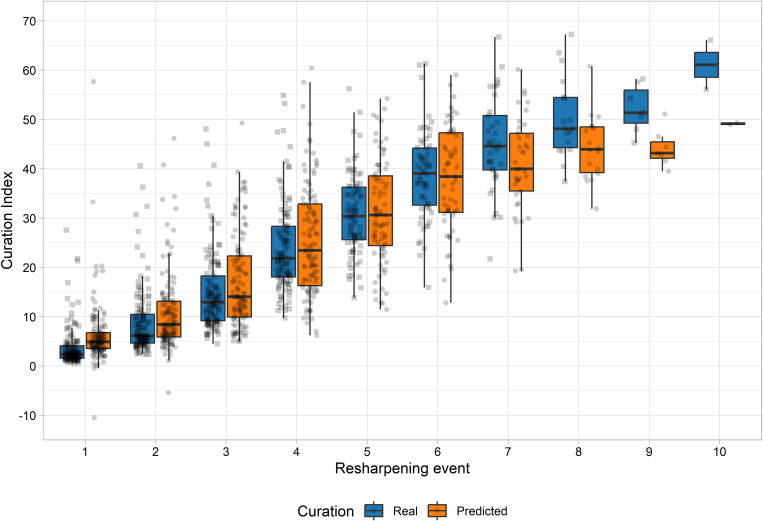
Actual and estimated curation values for each resharpening episodes.

Although knowing how many resharpening episodes an archaeological stone has undergone tool is unlikely, the availability of the data in a controlled experimentation allows for a better understanding of possible bias in the model. Fig 9 presents real and estimated values of curation according to each resharpening episode. Considering all episodes of retouch, no statistically significant difference is present between values of predicted and actual curation (t = 1.12, df = 1382.7; p = 0.262). However, a statistically significant difference is present between predicted and actual values of curation for the first episode of retouch (t = 3.811, df = 224.83; p < 0.001). This indicates that for scrapers which have undergone very light retouch, the random forest model will slightly overpredict their curation ratio. Statistically significant differences are also present between values of predicted and actual curation on flakes which have undergone eight or more resharpening episodes (t = −3.731, df = 48.493; p < 0.001). This means that scrapers which have undergone multiple episodes of retouch, with more than 50% of their mass removed, will have underpredicted curated ratios (Fig 9).

Table 5 presents the performance metrics values of the random forest model when leaving out 10% of flakes and all their resharpening episodes. Performance metrics present marginally lower values (Table 2). The Linear correlation ($r^{2}$) decreases from 0.974 to 0.96, MAE increases from 3.297 to 3.989, RMSE increases from 5.917 to 7.478, and MAPE increases from 6.775 to 7.929. When random forests are trained using only one random observation per flake (training set = 134) and the remaining resharpening episodes are used as test set (n = 560), there is a decrease on variance explained by the model ($r^{2}$ = 0.939), while the average error increases by 1.634 g (MAE = 4.931) and remaining dispersion error metrics also increase (RMSE = 9.242; MAPE = 10.503).

**Table 5:** Random forest performance metrics for calculating original mass when 10% of flakes and all their resharpening episodes are reserved as test set.

r^2^	MAE	RMSE	MAPE
0.96	3.989	7.478	7.929

**Fig 10:**
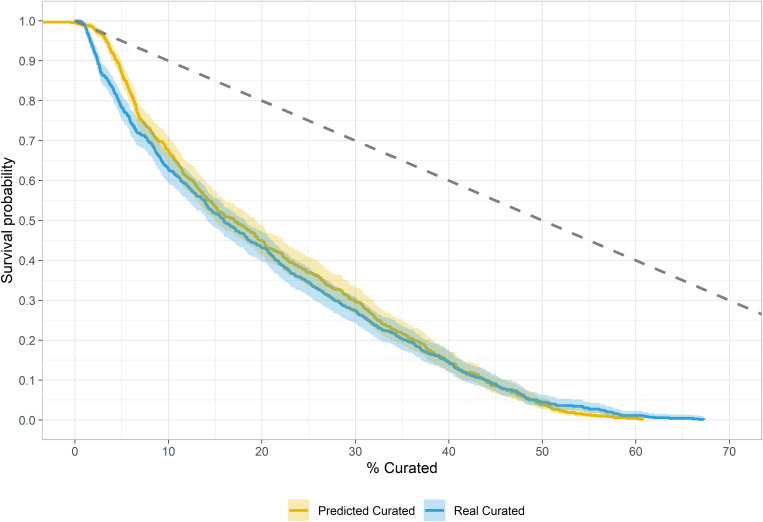
Survival curves of the actual and estimated values of curationn from the experimental sample.

Table 6 presents the results from fitting the Weibull distributions to the observed and estimated curated values of the experimental sample. The β and η estimates for the estimated curation are slightly higher (0.19 and 1.804) than the actual values, which would indicate a more prolonged use life. Fig 10 presents the results from fitting survivalship curves from both (actual and estimated) curated values. Both curves present Type III profiles overlapping for most of the distribution, although differences are seen at the start and end of the curves. At the start of the curve, estimated values indicate a higher survival probability. At the end of the curve, survival for actual curated values will extend beyond the 65% value, while the curve from estimated values ends slightly above the 60% value.

**Table 6:** Results from fitting Weibull distributions to the observed and estimated curation values.

Distribution	Beta	Scale
Observed curation	1.183	21.142
Estimated curation	1.373	22.946

When compared to other models using the same sample of flakes (Table 7), the random forest presents much better performance metrics. Neither of the two models presented linear correlation values ($r^{2}$) above the 0.8 threshold when predicting original flake mass.

**Table 7:** Performance metrics of alternative models trained using the same sample of 134 flakes.

Model	r^2^	MAE	RMSE	MAPE
Bustos-Perez & Baena, 2022	0.784	12.326	19.169	28.718
EPA, Platform, Max. Thick.	0.636	15.159	23.102	38.157

### 3.4 The “Original Scraper Mass Calculator” v.1.0.0

**Fig 11:**
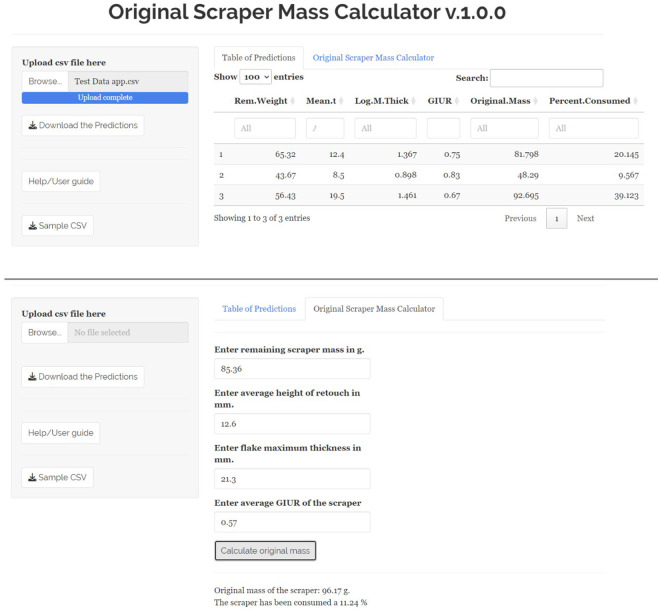
Screenshots of the Original Scraper Mass Calculator interface. Top: example of batch processing from a CSV file with a table of introduced data and its predictions. Bottom: individual introduction of data to calculate a scraper original mass.

In order to increase the applicability of machine learning models, the first version (v.1.0.0) of the original scraper mass calculator (OSMC) has been developed (Fig 11). This app integrates the above described random forest model into a user-friendly interface, allowing one to quickly estimate original scraper mass using the same set of variables. This app is published as a free open source Shiny app, allowing for its unrestricted use by the archaeological community.

The current version of the OSMC allows the user to individually make estimations of original scraper mass or to batch process large amounts of data. Individual estimations can be done by manually entering data of each scraper at their corresponding spots and pressing the “Calculate original mass” button. Processing of files with multiple entries requires uploading a CSV file containing the data and with correct names for each of the column variables (an example downloadable CSV file has been made available at the app to ease this process). Column names of the CSV file must be: Rem.Weight, Mean.t, Max.thick (log10 transformation of this variable is done automatically by the app), and GIUR. Both options return an estimation of the original scraper mass and what percentage has been lost through retouch. The OSMC has three levels of versioning numbering and it is published under v.1.0.0.

- v.0.0.1 corresponds to minor changes such as correction of parsing errors or minor changes in user interface.
- v.0.1.0 corresponds to medium changes, contemplating non-critical expansions of the existing data set (e.g.,: expansion of the data set to improve the training set data distribution), incorporation of new raw materials, adding of visualizations, inclusion of additional reports (e.g.,: indicator of quality of predictions). The overall performance metrics are not affected by these changes.
- v.1.0.0 corresponds to major changes, contemplating the implmentation of new models for predictions, critical expansions of the data set, deep modification of the user interface, incorporating new tool-types on which to make predictions (such as double scrapers, scrapers with ventral retouch, cores, etc.), modification (or giving the user the option to select) predictive variables.

The app is available at:

https://guillermo-bustos-perez.shinyapps.io/Original-Scraper-Mass-Calculator/

## 4. Discussion

The present study was aimed at predicting original scraper mass and from this the percentage of mass lost due to retouch (curation of an artifact). A set of 134 experimental flakes were sequentially retouched, and after each episode four variables were used as predictors. The set of variables were: scraper mass, maximum thickness of the flake (log10 transformation), average height of retouch, and the GIUR [23]. Four models were prepared using these variables: a multiple linear regression, a supported vector machine regression, a random forest, and a GBM. Random forest had the best performance metrics, both when predicting original scraper mass ($r^{2}$ = 0.974) and percentage of mass lost by retouch ($r^{2}$ = 0.839). VIF values from the multiple linear regression indicate that possible collinearity between predictors is of no consequence (all features presented VIF values bellow 10), and no statistically significant difference was found between the predictions of the complete model and the PCA model.

When 10% of flakes (n = 13) and all their resharpening episodes were used as test sets, the performance metrics only diminished marginally. It can be considered that this marginally lower performance is a result of a loss of information during training and not due to overfitting. The resulting model has been integrated into the first version of the “Original scraper mass calculator” in a user-friendly app, which allows random forest estimations from user data. Data can be batch processed using CSV files (columns should have same name as the ones from the model) or manually introduced. When the same random forest was trained using 134 random episodes per single flake and used to predict on the remaining resharpening episodes, the variance explained remained similar, and measures of error dispersion (RMSE, MAE and MAPE) did not increase drastically (it can be considered that the lower performance is a result of providing the model with less information). These results seem to indicate that the random forest is being able to adequately model the general patterns of flake reduction through retouch.

When the residuals from the complete model and an a model trained on the first two PC were compared, no statistical difference was found in their distribution, which can be considered as indicative of of an absence of overfit due to collinearity between predictors. However, results from training random forests without average height of retouch and GIUR resulted in similar performance metrics, indicating that the absence of one of these variables does not harm the overall performance of the model. For the final model of the OSMC all four variables have been kept. The following arguments have been considered when maintaining the four variables: first, the present model is already very simple, with only four predictive variables. While some collinearity is indicated by VIF (mostly structural collinearity resulting from calculating new variables from original predictors which will also be present in unseen data), it must be acknowledge that each of these variables are at different scales and act as proxies of different features. Average height of retouch presents values ranging from 0 to infinite, while GIUR ranges from 0 to 1. Average height of retouch works as a proxy of volume lost by retouch, while GIUR serves as a proxy of edge consumed by retouch. Thus, a strong argument can be made in order to remove one of both variables, or retain both (a common problem referred as feature selection). Second, distribution of residuals from the PCA did not differ from those of the complete model. Finally, the final model uses a random forest, which have been shown to be highly resistant to collinearity between predictors [53].

Scrapers with direct retouch on a continuous edge are some of the most common lithic implements present from the first Oldowan stone tools [1–3,82] through to modern ethnographic examples [4–7]. It is commonly considered that the amount of retouch that they receive is integral for understanding aspects of morphological variability and the organization of lithic technology by past societies [9,83–86]. Thus, being able to correctly estimate the amount and percentage of mass lost by retouch is fundamental for understanding these aspects of past human groups.

Most studies aimed at predicting original flake mass use complete flakes and not in the framework of a sequential resharpening experimentation. When predicting original flake mass, most studies presented linear correlation values raging between 0.224 to 0.750 [32,33,42,47]. Braun et al. [39] obtained a linear correlation value of 0.865 for an archaeological sample of materials when predicting the log of flake mass. Dibble and Pelcin [26] obtained an $r^{2}$ of 0.815 under experimental controlled conditions. Bustos-Pérez and Baena Preysler [30] obtained an $r^{2}$ of 0.813 for free hand-held knapped flakes after predicting log transformed values of flake mass and transforming them back to the to the linear scale. However, most of these studies worked with complete flakes, making it not possible to estimate the curation ratio on a sequential resharpening experimentation.

Studies evaluating the estimated reduction percentage (ERP) do provide accuracy metrics of predictions for original volume and percentage of volume lost by retouch for an experimental assemblage of flakes [21,24]. It is important to consider important differences between both experimental set-ups and the present research. ERP flakes were retouched only once, with most of them having lost less than 20% of volume due to retouch (less than 15% in the case of 3D ERP). When recorded manually [21], the ERP correlated very strongly with flake original volume ($r^{2}$ = 0.823) and fairly strong with percentage of volume lost by retouch ($r^{2}$ = 0.799 when outliers were removed). Incorporating a 3D protocol for applying the ERP [24] resulted in very strong correlation values for both estimates ($r^{2}$ = 0.891 when estimating original volume, and $r^{2}$ = 0.812 when estimating percentage of mass lost by retouch).

The higher correlation values obtained in the present study for original mass and percentage of mass removed by retouch can be attributed to four factors. First, the present study incorporated the scraper mass and maximum thickness. Scraper mass is probably helping machine learning algorithms model the minimum mass a scraper should have. The log transformed maximum thickness has been shown to serve as good proxy for original flake mass [37]. These two variables seem to be compensating for angle and length of retouch (used in the ERP and 3D-ERP), which would account for less than three percent of the variance when estimating original scraper mass for the given sample. Second, the present study also incorporated average height of retouch which strongly serves as a proxy of the amount of mass removed (and is also included in the ERP). Third, the present study used more robust regression methods, which are able to better model the provided variables and their interactions. This is especially noticeable in the results with tree-based methods (random forest and GBM) performing significantly better than the multiple linear regression. Fourth, tree-based methods are better at handling collinearity. Multiple linear regression and logistic regression fit coefficient estimates in order to make predictions. Collinear variables can result on unstable coefficients, which prevents the model from being applied to new data. Tree-based models use average values from data partition in order to make predictions. As a result of this, they handle collinearity better, with more stable predictions that can be generalized to new data, although the interpretation of variable importance might be hindered.

Weibull curves are a common approach when analyzing distribution of reduction among lithic assemblages [45,49,62,63]. The reliability of inferred Weibull estimates and survival curve shape will depend on three main factors. First, the ability of an index to accurately estimate reduction. This can be evaluated and compared through common model accuracy metrics (MAE, RMSE and MAPE). Second, that the accuracy of these estimates remains constant along the reduction process (accuracy of predictions will not change between a lightly and heavily consumed scraper). Third, that the accuracy of predictions is not affected by different ratios of use life and different values of original mass. Depending on size and shape, some flakes will undergo more resharpening episodes, allowing for high curation values along with high values of mass removed. An index might provide accurate estimates for flakes with high curation values reached after few retouch episodes (for example small backed flakes), but might struggle with initially big flakes which have undergone multiple episodes of retouch and reached high curation ratios. As a result of these three factors, the reliability of Weibull curves can be affected by how different indexes react to different scenarios of stone tool curation and original size. For example, an index which accurately estimates reduction among originally small flakes independent of their curation ratio will provide a reliable Weibull curve. However, the same index might struggle with originally bigger flakes which have undergone more retouch episodes and have higher curation ratios, thus resulting in unreliable Weibull curves. In the present study estimates for the Weibull distribution of observed and predicted curation ratios varied very slightly, with the same Type III of survival curves which only presented differences at the start (higher survival probability) and at the end (survival of actual values was slightly longer). This seems to indicate that, for the present experimental sample, the Weibull estimates and survival curves are reliable. However, the present research has made available the experimental dataset of all 134 flakes along with their original dimensions, resharpening episodes and common metrics used to develop reduction indices. This can help simulated different scenarios of stone tool curation and discard, and evaluate the reliability of different indexes when constructing Weibull curves.

A series of ideal qualities have been proposed for reduction indexes [56]. The random forest model presented here complies with the qualities of inferential power, directionality, comprehensiveness, sensitivity, and scale-independence. The model presents an extremely large/strong inferential power (>0.8) when estimating either original scraper mass or percentage of mass lost by retouch. This has allowed the model to make inferences at the individual level for specimens. Directionality, comprehensiveness and sensitivity are also present in the proposed model, with values of curation being detected from the beginning of retouch, and increasing with each episode of retouch. However, it is observed that there will be underestimations for big flakes which have lost large amount of mass by retouch (>50%).

A common drawback for estimating original scraper mass at the individual artifact level has been the obtention of negative values of curation (which happens when the predicted mass of the original flake blank is lower than the actual mass of the retouched scraper). This error was present in only three cases of the 698 mass predictions made by the random forest model. Although this error can still happen, it seems to be infrequent in the random forest model.

The random forest model is also scale-independent, since it can be applied to blanks of different sizes and it estimates the amount of mass lost from retouch as a percentage of the original mass. However, it is also important to point out that the inferences at the individual level allow for the model to obtain predictions at the linear scale (amount of mass in grams lost by an individual scraper), which can also be considered when analyzing lithic assemblages.

The random forest model complies with some of the qualities of blank diversity. Results from the present experimentation indicate that the predictions from the model are not affected by the different transversal section of flakes. The flat flake problem was specifically evaluated, with no statistically significant differences between residual distribution of flat and non-flat flakes. A statistically significant difference was also not present between both types of flakes on more advanced episodes of retouch. However, it still remains to be tested how the proposed index will perform on endscrapers made on blades. It is considered that on this tool-type the average height of retouch and GIUR will soon reach their maximum and remain stable, despite successive episodes of retouch.

The proposed model has limited versatility, since it can be only applied to scrapers with hard hammer direct retouch among a continuous edge. Although this type of scraper is fairly predominant among lithic assemblages, other tool-types such as double scrapers, scrapers with inverse or bifacial retouch, or scrapers retouched with soft hammer cannot be analyzed with the present model. Several limitations of the present research framework have already been indicated. As previously indicated, results show that very lightly retouched scrapers will have their curation ratio slightly overestimated, while heavily retouched scrapers will have a curation ratio underestimated. This bias at the lower an upper range of the predictions has been previously observed for random forests when predicting original flake mass [37]. In addition, and as previously stated, the application of the current model is limited only to scrapers with direct retouch on a continuous edge (excluding endscrapers made on blades, double scrapers or bifacially retouched scrapers). The initial experimental setting also presented a series of limitations. Values of original mass of scrapers presented a skewed distribution, with a long tail for flakes with a mass above 100 g. Additionally, data for flakes above 100 g is sparse, with few examples available. These two factors might be impacting accuracy of predictions for scrapers with higher values of initial mass, since the random forest and GBM have fewer examples from which to learn predictions.

Several additions can be done to expand and improve the current model in the future. As previously mentioned, the current model is limited to scrapers with direct retouch on one continuous edge. Further improvements using the current approach might benefit by adding “number of retouched edges” as a predictive variable or generating an independent new model specific for scrapers with multiple edges. Previous models predicting flake mass from remaining features [37] have shown that residual distribution might be affected by flake termination. Most flakes from the present experimental sample presented feather terminations. In the present work, the set of variables selected to predict flake mass differed regarding previous models, and it can be considered that variations of original mass induced by termination would be captured by remaining scraper mass. Further research expanding the experimental sample might help to systematically account for the effect of different termination types.

Parametric tests indicated a non-normal distribution of residuals from predicting original mass and curation ratio. Visual evaluation (Fig 7) of residuals when predicting flake mass according to predicted flake mass, indicate that the higher the predicted mass value, the residuals will become more disperse. However, this dispersion did not translate to the residuals of curation according to predicted flake mass. An underlying cause for the better distribution of curation residuals can is that the same error in estimating mass affects differently the estimation of curation on a small or big flake. An error of 3 g will translate into a much higher curation error for a flake with an original mass of 25 g, than a flake with an original mass of 80 g.

The current research tested four different machine learning algorithms (multiple linear regression, SVM regression, random forest and GBM). Random forest was the best performing algorithm although lightly retouched artifacts tend to have an overpredicted curation ratio, while extremely heavily retouched scrapers are usually underpredicted. Previous studies have shown that artificial neural networks (ANN) can improve upon random forests when predicting original flake mass [37]. Despite being computationally more expensive, further research might benefit from training ANN for predicting original scraper mass, helping to overcome the bias observed in lightly and extremely retouched artifacts. Other research focusing in core reduction intensity have predicted curation ratio through a logistic error distribution model [43], which avoids obtaining negative percentages of reduction intensity (predicted mass has a lower value than remaining mass). This would offer the possibility of predicting mass from curation, instead of curation from mass, although comparing accuracy metrics between both methods is required. The “Original Scraper Mass Calculator” has an initial versioning number of v.1.0.0. Three levels of versioning number have been implemented. The underlying idea of the versioning number is the dynamic conception of the developed application. This dynamic conception can help to remedy possible deficiencies through the expansion of the current dataset. Furthermore, it also allows to expand the dataset in order to incorporate new raw materials, tool types, or even by training new models that will help improve the quality of predictions (for example replacing the random forest model for a more accurate model, or changing the approach of estimating curation ratiois contemplated as a major change). This dynamic approach allows for flexibility when updating the Original Scraper Mass Calculator.

The v.1.0.0 of the “Original Scraper Mass Calculator” does not provide indications of how reliable predictions are. Very lightly retouched artifacts tend to have an overestimated curation ratio, although predictions might be reliable. However, caution is recommended when it is inferred that a scraper has undergone multiple episodes of retouch since predictions from the current model will tend to underestimate the real amount of mass removed by retouch. Detecting cases of underestimation currently depends on the user knowledge of their lithic assemblage. An experienced lithic analyst will be able to detect cases of underestimation based on known features which determine flake mass (platform depth and size, flake thickness, etc.). Further versions of the “Original Scraper Mass Calculator” can include an indicator of the quality of predictions based on values of the GIUR index, or using an interaction of the GIUR and maximum thickness (allowing it to detect big/thick flakes which have undergone multiple episodes of resharpening).

Accurate inferences of mass lost by individual scrapers can be combined with multiple lithic analysis. Original scraper mass and the curation ratio can be combined with geometric morphometrics to determine how scraper shape changes through reduction (allometry) at a single (or multiple) sites, or at a diachronic level [8,45,87–92]. Distance to raw material sources seems to have played an underlying factor of resharpening intensity among scrapers, with scrapers coming from longer distances having been resharpened more intensively [93–97]. However, concerns about accuracy of indexes employed for these estimations have been raised [21,24]. Application of the proposed method can help better model the influence of raw material distance on resharpening intensity. Association between resharpening intensity, tool shape and use remain partially unexplored. Some studies have pointed to a lack of relationship between specific tool form and function [98], while other studies point at potential higher functional versatility for tools coming from longer distances and higher curation ratios [99]. Finally, the application of the proposed method can help to better model two additional aspect of lithic technological organization: changes in preferred blanks for resharpening and changes in tool gear transport. Changes in preferred initial blanks for resharpening have been observed between different Paleolithic periods but also within a same Paleolithic period. A clear example is observed among the different lithic technocomplexes of the western European Middle Paleolithic. Different lithic technocomplexes, characterized by different predominant knapping methods, show differences in the selection of initial blanks which underwent more intense resharpening [20,86,100]. Additionally, strategies of tool transport also experienced changes within technocomplexes, alternating between the transport and resharpening of tools and transport of cores for immediate flake production [83,101].

## 5. Conclusions

Predicting original scraper mass and amount of mass lost by retouch has long been a major goal in lithic analysis. An experimental sample of 134 flakes was sequentially retouched, and a new combination of variables (scraper mass, average height of retouch, maximum thickness, and value of the GIUR index) was recorded for each resharpening episode. This new set of variables in combination with more robust regression algorithms has resulted in the most accurate model to date. This higher accuracy allows for an estimation of retouch intensity at the individual scraper level. This model has been integrated into a user-friendly app in order to allow for its widespread application among the archaeological community.
